# A SARS-CoV-2 sequence submission tool for the European Nucleotide Archive

**DOI:** 10.1093/bioinformatics/btab421

**Published:** 2021-06-07

**Authors:** Miguel Roncoroni, Bert Droesbeke, Ignacio Eguinoa, Kim De Ruyck, Flora D’Anna, Dilmurat Yusuf, Björn Grüning, Rolf Backofen, Frederik Coppens

**Affiliations:** 1 Department of Plant Biotechnology and Bioinformatics, Ghent University, 9052 Ghent, Belgium; 2 VIB Center for Plant Systems Biology, 9052 Ghent, Belgium; 3 Department of Computer Science, University of Freiburg, 79110 Freiburg im Breisgau, Baden-Württemberg, Germany

## Abstract

**Summary:**

Many aspects of the global response to the COVID-19 pandemic are enabled by the fast and open publication of SARS-CoV-2 genetic sequence data. The European Nucleotide Archive (ENA) is the European recommended open repository for genetic sequences. In this work, we present a tool for submitting raw sequencing reads of SARS-CoV-2 to ENA. The tool features a single-step submission process, a graphical user interface, tabular-formatted metadata and the possibility to remove human reads prior to submission. A Galaxy wrap of the tool allows users with little or no bioinformatics knowledge to do bulk sequencing read submissions. The tool is also packed in a Docker container to ease deployment.

**Availability and implementation:**

CLI ENA upload tool is available at github.com/usegalaxy-eu/ena-upload-cli (DOI 10.5281/zenodo.4537621); Galaxy ENA upload tool at toolshed.g2.bx.psu.edu/view/iuc/ena_upload/382518f24d6d and github.com/galaxyproject/tools-iuc/tree/master/tools/ena_upload (development); and ENA upload Galaxy container at github.com/ELIXIR-Belgium/ena-upload-container (DOI 10.5281/zenodo.4730785).

## 1 Introduction

The current COVID-19 pandemic caused by the SARS-CoV-2 virus has highlighted the importance of open and FAIR (Findable, Accessible, Interoperable, Reusable) (Wilkinson *et al.*, 2016) data. Fast and open access to all types of data (biomolecular, epidemiological, societal) is paramount to the quick development and deployment of tests, vaccines, treatments and policies used in the fight against the virus.

Genome sequences of SARS-CoV-2 are available since early January 2020 (Wu *et al*., 2020) and have enabled, among other things, the design of polymerase chain reaction tests and vaccines. Currently, genome sequencing is the only way for detecting and monitoring new, and potentially more infectious, virus variants. In the COVID-19 Data Portal (www.covid19dataportal.org), the repository for SARS-CoV-2 nucleotide sequence data is the European Nucleotide Archive (ENA). ENA stores raw sequencing data, assemblies and annotation data (www.ebi.ac.uk/ena/browser/home). As part of the International Nucleotide Sequence Database Collaboration (INSDC), ENA also indexes data from the NCBI and DDBJ (Arita *et al.*, 2021).

The Global Initiative on Sharing All Influenza Data (GISAID, www.gisaid.org) is a specialized repository for genetic, clinical and epidemiological data of influenza viruses and coronaviruses. GISAID is also one of the data resources mentioned in the European guidelines for open access to COVID-19 data. It differs from ENA and other INSDC repositories in some key aspects: access to data and data submission are only possible after application and registration; reuse of data is restricted; and only the consensus sequence of assembled genomes is accepted.

Raw reads contain valuable information, such as coverage depth and quality scores, that is lost in a consensus sequence. Variant callers with different stringencies on coverage and quality may produce slightly different consensus sequences from the same raw reads (Black *et al.*, 2020). Accurate and reproducible variant calling is particularly important for the detection and monitoring of new virus variants.

Submissions to both GISAID and ENA are increasing exponentially. However, while over 1.7 M SARS-CoV-2 genome sequences have been submitted to GISAID, only 645 K raw reads submissions occurred in ENA-INSDC as of May 2021. This has two important implications for the research community: the majority of SARS-CoV-2 genomes are not fully FAIR and their underlying raw data remains unpublished. We believe that this discrepancy is in part caused by the technical barrier to submitting large amounts of raw reads to ENA which requires command line knowledge and metadata in XML format, putting off many researchers and clinicians.

We have developed a tool to facilitate the submission of SARS-CoV-2 raw sequence reads to ENA. The tool simplifies the submission process by performing ’Study’ and ’Sample’ registration, and data submission in one step. Metadata is submitted via a single spreadsheet template containing all mandatory fields and controlled vocabulary or interactively. The tool is available in the command line, as a Galaxy tool, and packed in a Docker container. There are many advantages of porting this submission tool to Galaxy including a graphical user interface, access to tools and workflows (including SARS-CoV-2-specific ones) (Maier *et al.*, 2021) for preprocessing, downstream analysis and visualization of sequences. Galaxy also provides a platform for sharing of data and metadata, facilitating international collaboration, integration with other public resources and enabling publishing FAIR data and analysis workflows.

**Fig. 1.**
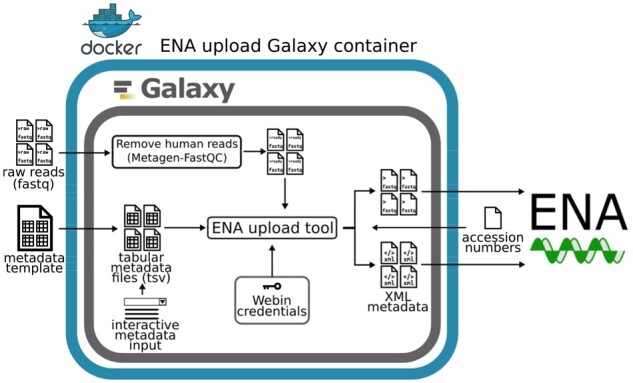
Schematic data and metadata flow of a submission of raw reads to ENA using the ENA upload Galaxy container. Raw reads can be processed with Metagen-FastQC tool to remove human reads. Metadata can be input using a template spreadsheet or interactively in Galaxy. Webin credentials are kept in the Galaxy user profile. Upon execution, sequence files are uploaded to ENA FTP server and ’Study’ and ’Sample’ are registered. Accession numbers are retrieved, added to the metadata and this is pushed with cURL to ENA to complete the submission

Figure 1 outlines the data and metadata flow using the different instances of the tool.

## 2 Features

### 2.1 Command line interface ENA upload tool

This python script is used for programmatic submissions of sequencing reads to ENA. It has a command line interface and requires four metadata tables, the sequence files and a Webin ID and password. Metadata is imported through four tabular (tsv) files, one each for ’Study’, ’Sample’, ’Experiment’ and ’Run’ (ena-docs.readthedocs.io/en/latest/submit/general-guide/metadata.html). From these tables, the tool uses the Genshi template engine to generate XML files needed for a programmatic submission of reads to ENA, including the submission XML file. The tool submits the sequence data to the user’s private Webin file upload area at EMBL-EBI using FTP and uses the checksum of the data files to ensure the data integrity. To perform bulk submission of all objects, the aliases IDs in different ENA objects should correspond to the aliases IDs in the experiment object, linking all objects together. After the data files are submitted, the tool registers the ’Study’, ’Samples’, ’Experiments’ and ’Runs’ metadata at ENA. Submission of the XML metadata is done with cURL.

### 2.2 Galaxy ENA upload tool

The command line submission tool was also wrapped into a Galaxy tool. Galaxy’s graphical user interface makes it more accessible to researchers with limited or no command line experience. Additionally, Galaxy offers a myriad of tools and workflows to preprocess and analyze genetic sequence data, including COVID-19 specific workflows (covid19.galaxyproject.org/). The user adds the Webin credentials required for submission to the Galaxy user profile. For custom Galaxy instances, the admin can enable users to set their own credentials using a yaml configuration file. In addition to the tsv files, the user can input the metadata interactively in Galaxy or complete and upload a metadata template spreadsheet. In all cases, the Galaxy submission tool converts the metadata to the tsv files required to generate the xml files for submission. The tool is indexed at Galaxy’s tool shed and available for use at usegalaxy.eu. The ENA upload tool is part of the Intergalactic Utilities Commission, a curated collection of Galaxy tools.

### 2.3 ENA upload Galaxy container

We made use of the Galaxy Docker base image (github.com/bgruening/docker-galaxy-stable) to pack the submission tool into a custom-tailored ENA upload Galaxy container. The Galaxy instance in the container has a user guide in its middle pane and includes a tool to remove contaminant human reads. This tool is a wrap of Metagen-FastQC that removes any reads that map to the human genome (github.com/Finn-Lab/Metagen-FastQC). Mapping is done using BWA-MEM (Li and Durbin, 2010) and can be resource intensive. BWA-MEM took an average of 39.0 min (3.0 min SD) and 5.26 GB of memory (0.01 GB SD) to align one Ion Torrent sequence file (*n* = 8, mean file size was 182 MB) on a workstation with 8 GB memory and 1.6 GHz eight-core processor. The whole container running the cleanup tool peaked at 6.23 GB memory (0.19 GB SD). We recommend this tool for containers deployed on clusters with ample memory resources. The human reference genome is stored in a CernVM-FS repository. A FUSE client is mounted upon running the container to be able to access the repository. An alternative version of the container includes a copy of the indexed human reference genome hg38. Included is also a workflow to preprocess short reads (Illumina or ONT) before submission, adapted from an existing Galaxy SARS-CoV-2 preprocessing workflow (github.com/galaxyproject/SARS-CoV-2/tree/master/genomics). Installing the dependencies for this workflow is done automatically using the workflow file.

## 3 Implementation

The ENA upload Galaxy container was used by ELIXIR Belgium to submit SARS-CoV-2 sequence data from Belgian patients to ENA. We submitted 39 sequences (runs) from 35 samples obtained from oropharyngeal swabs of COVID-19 patients at Hospital AZ Rivierenland in Antwerp (PRJEB40711). The data were provided by the Institute of Tropical Medicine (www.itg.be). Additionally, eight SARS-CoV-2 sequences from eight postmortem FFPE lung tissue samples (Hôpital Erasme-Université Libre de Bruxelles) were successfully submitted to ENA (PRJEB42699).

## 4 Conclusion

In keeping with FAIR principles, the ENA offers unrestricted access and reuse of raw and analyzed sequence data. The set of tools presented here facilitate the submission of raw SARS-CoV-2 sequences to ENA. It should prove particularly useful for users submitting large numbers of raw read data files programmatically and/or for users with limited or no bioinformatics skills. Submitting raw viral sequence data to ENA ensures that all researchers can access and use this in the response against COVID-19.

## Funding

This work was supported by Fonds Wetenschappelijk Onderzoek [I002819N] and Sonderforschungsbereich/TRR [167/2 Z01].

*Conflict of Interest*: none declared.

## References

[btab421-B1] Arita M. et al (2021) The international nucleotide sequence database collaboration. Nucleic Acids Res., 49, D121–D124. 33166387 10.1093/nar/gkaa967 PMC7778961

[btab421-B2] Black A. et al (2020) Ten recommendations for supporting open pathogen genomic analysis in public health. Nat. Med., 26, 832–841. 32528156 10.1038/s41591-020-0935-z PMC7363500

[btab421-B3] Li H. , Durbin R. (2010) Fast and accurate long-read alignment with Burrows-Wheeler transform. Bioinformatics, 26, 589–595. 20080505 10.1093/bioinformatics/btp698 PMC2828108

[btab421-B4] Maier W. et al (2021) Freely accessible ready to use global infrastructure for SARS-CoV-2 monitoring. bioRXiv, doi:10.1101/2021.03.25.437046.

[btab421-B5] Wilkinson M. et al (2016) The FAIR guiding principles for scientific data management and stewardship. Sci. Data, 3, 160018. 26978244 10.1038/sdata.2016.18 PMC4792175

[btab421-B6] Wu F. et al (2020) A new coronavirus associated with human respiratory disease in China. Nature, 579, 265–269. 32015508 10.1038/s41586-020-2008-3 PMC7094943

